# 

**Published:** 1845-04

**Authors:** 


					﻿THE
WESTERN JOURNAL
OF
MEDICINE AND SURGERY.
editors.
DANIEL DRAKE, M. D.,
PROFESSOR OF PATHOLOGY AND THE PRACTICE OF MEDICINE
IN THE MEDICAL INSTITUTE OF LOUISVILLE:
LUNSFORD P. YANDELL, M. D„
PROFESSOR OF CHEMISTRY AND PHARMACY IN THE SAME:
THOMAS W. COLESCOTT, M. D.
BOOKS RECEIVED.
The Cyclopaedia of Practical Medicine is completed, and is al-
ready on sale in some of the bookstores in four large volumes.
Those who have not subscribed for the work have now the opportu-
nity of getting it entire. It is what it claims to be, a cyclopaedia,
in which practical medicine is posted up to the present day, and as
such constitutes a store-house of medical knowledge upon which
the student and practitioner may draw with equal advantage.
We have received only the first number of Copland’s Dictionary.
We hope the publication has not been suspended.
We are indebted to Professor Spencer, of Geneva College, for a
copy of his lecture on Animal Heat, in a well printed little volume
of 114 pages. We shall take great pleasure in presenting to our
readers an analysis of these lectures in some future number. Y.
				

